# Comparative dose-response study on the infusion of norepinephrine combined with intravenous ondansetron versus placebo for preventing hypotension during spinal anesthesia for cesarean section: a randomised controlled trial

**DOI:** 10.1097/JS9.0000000000000920

**Published:** 2023-11-23

**Authors:** Zhi-min Sheng, Heng-qiu Sun, Jun-qin Mao, Jie Liu, Gang Liang, Zhong Mei

**Affiliations:** a Department of Anesthesiology, Wenling Maternity and Child Health Care Hospital, Taizhou, China; b Department of Pediatric Surgery, Taizhou Women and Children’s Hospital of Wenzhou Medical University, Taizhou, China; c Department of Anesthesiology, Affiliated Xiaoshan Hospital, Hangzhou Normal University, Hangzhou, China

**Keywords:** Dose-response, hypotension, norepinephrine, ondansetron, spinal anaesthesia

## Abstract

**Background:**

Ondansetron has been reported to attenuate the incidence of spinal anaesthesia-induced hypotension (SAIH) and norepinephrine requirement during caesarean section. However, no quantitative study has evaluated the extent of this effect. This study aimed to determine the dose-response of prophylactic infusion of norepinephrine to prevent SAIH in parturients who received intravenous ondansetron or placebo before spinal anaesthesia for caesarean section. The median effective dose (ED_50_) and 90% effective dose (ED_90_) were compared to evaluate the effect of ondansetron versus placebo on the norepinephrine requirement.

**Materials and methods:**

One hundred fifty parturients undergoing caesarean section were randomized to receive either 0.1 mg/kg ondansetron (group O) or saline control (group C) 10 min before spinal anaesthesia. The parturients were randomly assigned to one of five different norepinephrine infusion groups: 0.02, 0.04, 0.06, 0.08 or 0.10 µg/kg/min. An effective infusion dose of norepinephrine was defined as non-occurrence of hypotension during the study period. The values for ED_50_ and ED_90_ of norepinephrine infusion were determined using probit regression. Differences between the two groups were evaluated by comparing the relative median potency with 95% CIs.

**Results:**

The ED_50_ values were 0.033 (95% CIs, 0.024–0.043) µg/kg/min in group C and 0.021 (95% CIs, 0.013–0.029) µg/kg/min in group O. The ED_90_ values were 0.091 (95% CIs 0.068–0.147) µg/kg/min in group C and 0.059 (95% CIs 0.044–0.089) µg/kg/min in group O, respectively. The estimate of the relative median potency for norepinephrine in group C versus group O was 0.643 (95% CIs, 0.363–0.956). The incidence of side effects was comparable between groups. No significant difference in neonatal outcomes.

**Conclusion:**

Intravenous ondansetron 0.1 mg/kg before spinal anaesthesia significantly reduced the dose requirement of prophylactic norepinephrine infusion in parturients undergoing elective caesarean section. This finding is potentially useful for clinical practice and further research.

## Introduction

Highlights

- No quantitative study has evaluated the effect of ondansetron on the prophylactic infusion of norepinephrine in parturients undergoing caesarean section.
- Intravenous ondansetron 0.1 mg/kg before spinal anaesthesia significantly reduced the dose requirement of prophylactic norepinephrine infusion in parturients undergoing elective caesarean section.
- The results of this study provide guidance for the initial infusion dose of norepinephrine with or without prophylactic intravenous ondansetron to prevent hypotension during spinal anaesthesia for caesarean section.

Spinal anaesthesia-induced hypotension (SAIH) is a major complication during caesarean section, resulting in many detrimental effects for both the mother and neonate^1,2^. As vasopressors are considered among other effective strategy for preventing SAIH^3,4^. Although phenylephrine is commonly used in obstetric anaesthesia, its significant effect on maternal heart rate (HR) and cardiac output (CO) has been questioned^5,6^. Norepinephrine as a potent α-adrenergic receptor agonist also stimulates β-adrenergic receptors, which significantly reduces bradycardia and better maintains CO, thereby providing a potential alternative to phenylephrine^2,7^.

The reduction in systemic vascular resistance by blockade of sympathetic fibres is a major cause of SAIH^8^. Activation of Bezold Jarish reflex (BJR) after spinal anaesthesia induces vasodilatation and reduces venous return leading to hypotension and bradycardia^9^. Some studies have illustrated that serotonin (5-HT) subtype 3 (5-HT3) receptors play a crucial role in the occurrence of the BJR^10,11^, and BJR can be blocked by 5-HT3 antagonists^12^. Ondansetron, a widely used 5-HT3 receptor antagonist, has been reported to attenuate the incidence of SAIH and vasopressor consumption in obstetric^13,14^ and non-obstetric patients^9,15^.

Although one study found that intravenous ondansetron 4 mg before spinal anaesthesia reduced the median effective dose (ED_50_) of prophylactic phenylephrine infusion by approximately 26% in parturients undergoing caesarean section^16^. No quantitative study has evaluated the effect of ondansetron on the prophylactic infusion of norepinephrine in parturients undergoing caesarean section. Reducing the requirement for norepinephrine may be clinically useful because of concerns regarding the possible adverse effects of norepinephrine extravasation through the peripheral vein^17,18^. Excessive infusion time or dosage can also lead to a reduction in renal perfusion, thereby contributing to renal failure^19^. Furthermore, the majority of current studies have utilized fixed doses of ondansetron. Given the large variation in maternal weight among different populations, we believe that a weight-adjusted dose of ondansetron may be more suitable for clinical practice.

The main aim of this study was to determine the dose-response of prophylactic norepinephrine infusion for preventing SAIH in parturients who received a single dose of 0.1 mg/kg intravenous ondansetron or placebo prior to spinal anaesthesia for caesarean section. The ED_50_ and 90% effective dose (ED_90_) values were compared to evaluate the extent of the effect of ondansetron on norepinephrine requirement compared with placebo.

## Materials and methods

### Study design

This placebo-controlled, double-blind, randomized, dose-finding trial was conducted strictly in compliance with the principles of the Helsinki Declaration from March 2021 to May 2022, and was approved by the Institutional Review Board of Wenling Maternity and Child Health Care Hospital. (No. 2020-IRB-003) on 9 November 2020. The work was reported in line with the Consolidated Standards of Reporting Trials (CONSORT) guidelines^20^. All the parturients enrolled in this trial signed a written informed consent form. The trial was registered at Chictr.org.cn ( No. Chi CTR 2000040295) on 27 November 2020.

### Study population

A total of 168 American Society of Anesthesiologists (ASA) physical status less than III parturients with uncomplicated term pregnancies (≥37 wk), aged 18–40 years, and scheduled for elective lower-segment caesarean section under spinal anaesthesia were enroled. The exclusion criteria were allergy to ondansetron or norepinephrine, contraindications to regional anaesthesia, hypertensive disorders of pregnancy, cardiovascular disease, diabetes mellitus, multiple gestation, obesity (BMI≥35 kg/m^2^), height less than 145 or greater than 175 cm, placenta previa, ruptured membranes and significant coexisting maternal disease (such as severe anaemia, asthma, hyperthyroidism, and significant liver disease).

### Study procedures and interventions

Parturients were randomly allocated to group O (ondansetron) or group C (saline control) in a 1:1 ratio using MedCalc for Windows (version 18.2.1, Ostend, Belgium), and separate randomization code sequences were created for the two groups. Then the parturients were randomly assigned to 1 of 5 different prophylactic infusion groups of norepinephrine: 0.02, 0.04, 0.06, 0.08 or 0.10 µg/kg/min (15 per subgroup). Subsequently, the codes were secured in sequentially numbered, individually sealed opaque envelopes.

On the day of surgery, a research nurse who was not involved in case management prepared a batch of identical 10 ml syringes labelled study drug which containing 0.1 mg/kg ondansetron diluted in normal saline to 10 ml or 10 ml normal saline (placebo). The dosage basis of ondansetron was based on a published article^21^ and our pilot study, in which 0.1 mg/kg ondansetron was effective in reducing the prophylactic norepinephrine infusion. She also prepared a batch of identical 50 ml syringes containing dilute solutions of different concentrations of norepinephrine according to randomization. The dosage of norepinephrine for each group was calculated according to the following formulae: weight (kg)×1.2 µg/kg in group 0.02; weight (kg)×2.4 µg/kg in group 0.04; weight (kg)×3.6 µg/kg in group 0.06; weight (kg)×4.8 µg/kg in group 0.08; and weight (kg)×6.0 µg/kg in group 0.10. They were then handed over to the attending anesthesiologist who recorded the intraoperative data and administered norepinephrine at a rate of 50 ml/h. The attending anesthesiologist was blinded to the actual concentration of norepinephrine administered to the parturients.

Upon entering the operating room, all the parturients were placed on the operating table in the supine position. An indwelling 18-gauge intravenous catheter was inserted into the right forearm vein and no fluid was preloaded. Routine monitoring, including electrocardiography, noninvasive blood pressure (NIBP) and pulse oximetry was conducted. Baseline systolic blood pressure (SBP) and HR were measured by calculating the mean of three consecutive measurements at 2-min intervals after a brief rest. Parturients were randomized to receive intravenously either 0.1 mg/kg ondansetron (group O) or saline control (group C) 10 min before spinal anaesthesia, administered by a research assistant not involved in data collection and intraoperative management. The combined spinal-epidural anaesthesia was performed at the L2–L3 or L3–L4 interspace with 16 mg of hyperbaric ropivacaine at a rate of 0.2 mL/sec after confirmation of free flow of clear cerebrospinal fluid by an anesthesiologist using a needle-through-needle technique with parturients in a left lateral position, and the parturient was then placed in a supine position with left uterine displacement of approximately 15° by means of a wedge. No drugs were administered through the epidural catheter.

Concurrent with spinal anaesthesia, a volume of 10 ml/kg of lactated Ringer’s solution was infused within 10 min. While the intrathecal injection was administered, prophylactic norepinephrine infusion was initiated. NIBP and HR were recorded every one minute until the time of delivery and then cycled every three minutes until the end of the surgery. Surgery was permitted when the sensory block reached the T6 level.

Hypotension (defined as a decrease in SBP≥20% from baseline) and severe hypotension (defined as a decrease in SBP≥40% from baseline) were treated with an intravenous bolus of 6 µg norepinephrine, which was repeated if SBP failed to return above 90% of baseline. The definition of hypertension was an increase in SBP≥20% from baseline, which was managed by stopping norepinephrine infusion. When the SBP decreased to near the baseline SBP, the infusion was restarted. HR less than 50 beats/min was defined as bradycardia. If it was accompanied by hypotension, atropine (0.5 mg) was administered. If not accompanied by hypotension, the infusion of norepinephrine was suspended.

### Outcome measure

The study period was defined as the duration between the initiation of spinal injection and the delivery of the foetus. An effective dose of norepinephrine infusion was defined as non-occurrence of hypotension during this period. The primary endpoint was the incidence of SAIH at different infusion doses during the study period. Secondary endpoints included demographic characteristics (such as age, height, weight, sensory block level, surgical data, and total norepinephrine consumption), and incidence of side effects (hypotension, severe hypotension, hypertension, nausea, vomiting, bradycardia and shivering). Neonatal outcomes (arterial blood gas analysis, Apgar scores and birth weight) were also recorded.

### Sample size estimation

The sample size design was based on data from previously published studies, which showed that the ED_50_ and ED_90_ values for prophylactic infusion of norepinephrine combined with crystalloid coload were approximately in the range of 0.03-0.10 µg/kg/min^22,23^. Therefore, we decided to divide the parturients into two main groups with five subgroups each (0.02, 0.04, 0.06, 0.08 or 0.10 µg/kg/min).

The sample size was calculated with the Cochran–Armitage Test using PASS (Version 11.0.1; NCSS, LLC). According to data from our published study^22^, the percentages of parturients with effective prevention of SAIH were 0.40, 0.55, 0.70, 0.90, and 0.95 in parturients (20 per subgroup) who received prophylactic norepinephrine infusions at rates of 0.02, 0.04, 0.06, 0.08 and 0.10 µg/kg/min respectively. A sample size of 10 parturients per subgroup was required to provide the trial with 90% power to detect a linear trend among the groups with SAIH using a Z-test with continuity correction and a significance level of 0.05. Considering the narrowing CIs and potential dropouts, we planned to enrol 75 parturients per main group (15 per subgroup).

### Statistical analysis

Statistical analyses were performed using Graphpad Prism Statistical software (version 8.0.2 Inc) and SPSS 25.0 for Windows (IBM Corp). *P* values less than 0.05 were considered significant. The normality of data distribution was assessed using the Kolmogorov–Smirnov test, and continuous variables were expressed as mean±standard deviation (SD) and median [range] when appropriate. The normally distributed data were compared using the independent Student *t*-test. The non-normally distributed data were compared using the Mann–Whitney U-test. The chi-square test was used for comparisons of frequency data and presented as number (%). The values for ED_50_ and ED_90_ with 95% CIs of the prophylactic norepinephrine infusion dose were determined using probit regression. Differences between the two groups were evaluated by calculating the relative median potency with 95% CIs.

## Results

**Figure 1:**
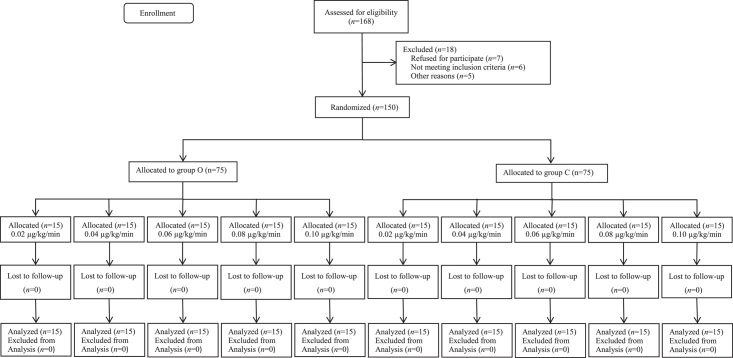
Consolidated standards of reporting trials diagram showing patient recruitment and flow.

**Figure 2:**
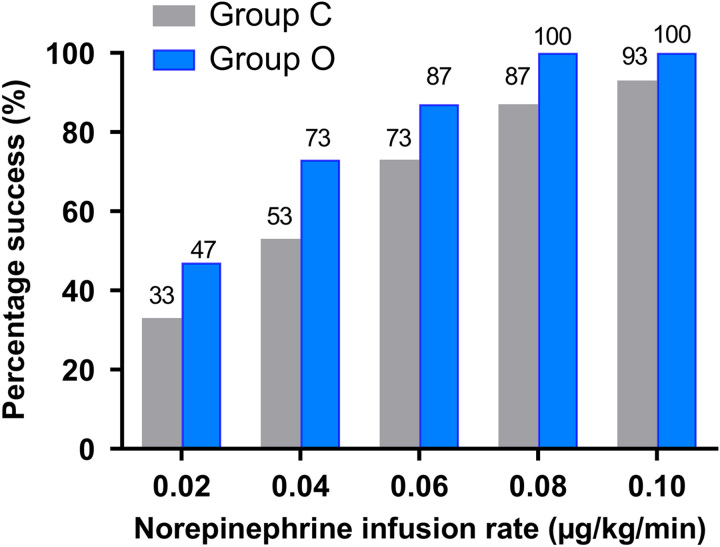
The success rate of preventing hypotension at different infusion rate of norepinephrine. This bar chart differentiates success rate of the grey bar (Group C) from the solid blue bar (Group O) (success was defined as systolic blood pressure greater than or equal to 80% of the baseline value after spinal anaesthesia). Group C=saline control group, Group O=ondansetron group.

A total of 168 parturients were recruited for this study. And 18 parturients were excluded from the study. Detailed information is provided in the consort diagram in Figure 1. No statistically significant differences were found in the demographic data, surgical duration, sensory block level, total norepinephrine consumption (before delivery), and intravenous fluid volume between the two groups (Table 1). Side effects, such as hypotension, severe hypotension, hypertension, nausea, vomiting, bradycardia, and shivering were comparable among the groups (Table 2). The success rate of different infusion doses of norepinephrine for preventing hypotension in the two groups is shown in Figure 2. Norepinephrine doses of 0.02, 0.04, 0.06, 0.08 or 0.10 µg/kg/min corresponded to successful prevention of SAIH by 33%, 53%, 73%, 87%, and 93% in group C, and 47%, 73%, 87%, 100%, and 100% in group O, respectively.

**Table 1:** Demographic data and anaesthesia related data.

	Group O (*n*=75)	Group C (*n*=75)	*P*
Age (year)	30.2±4.8	30.4±4.9	0.827
Height (cm)	158.7±4.2	158.8±4.3	0.862
Weight (kg)	71.2±8.7	72.0±9.6	0.605
Gestational age (week)	38.9±1.0	39.0±1.0	0.531
Baseline SBP (mmHg)	118.9±8.9	118.1±8.7	0.587
Baseline HR (bpm)	88.1±9.7	89.2±11.1	0.506
Sensory block level (T)	T4 [T4–T5]	T4 [T4–T5]	0.633
Spinal anaesthesia to delivery interval (min)	17.9±3.0	17.1±3.0	0.109
Time from administration of ondansetron to spinal injection (min)	14 [13–16]	14 [12–15]	0.415
Total norepinephrine consumption (before delivery) (µg)	75.3±33.6	85.5±42.6	0.106
Intravenous fluid volume given (before delivery) (ml)	711.9±86.8	719.6±95.9	0.605

Data are presented as mean±SD, or median [interquartile range].

HR, heart rate; SBP, systolic blood pressure.

**Table 2:** Hemodynamic changes, side effects.

	Group O (*n*=75), *n* (%)	Group C (*n*=75), *n* (%)	*P*
Hypotension (before delivery)	14 (18.7)	24 (32.0)	0.060
Severe hypotension (before delivery)	2 (2.7)	5 (6.7)	0.246
Hypertension (before delivery)	4 (5.3)	3 (4.0)	0.699
Nausea	6 (8.0)	9 (12.0)	0.414
Vomiting	1 (1.3)	3 (4.0)	0.311
Bradycardia	1 (1.3)	2 (2.7)	0.560
Shivering	5 (6.7)	6 (8.0)	0.754

Data are presented as number (%). Categorical data were analyzed using the Cochran–Armitage χ2 test for trend. Hypotension was defined as systolic blood pressure<80% of baseline value. Severe hypotension was defined as systolic blood pressure<60% of baseline value. Reactive hypertension was defined as systolic blood pressure>120% of baseline value. Bradycardia was defined as HR<50 beats/min.

HR, heart rate.

**Figure 3:**
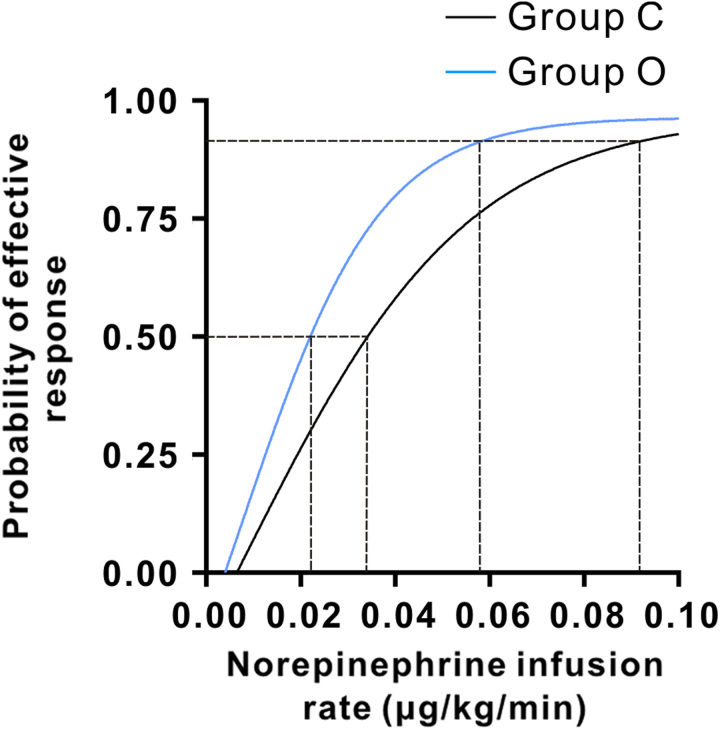
Dose-response curve of norepinephrine infusion combined with ondansetron versus placebo for preventing hypotension plotted from estimated probabilities of effective response calculated using probit regression. Group C=saline control group, Group O=ondansetron group.

The dose-response curves for the dose of norepinephrine infusion for preventing SAIH were determined using probit regression analysis and presented in Figure 3. The ED_50_ values were 0.033 (95% CIs, 0.024–0.043) µg/kg/min in group C and 0.021 (95% CIs, 0.013–0.029) µg/kg/min in group O. The ED_90_ values were 0.091 (95% CIs 0.068–0.147) in group C and 0.059 (95% CIs 0.044–0.089) µg/kg/min in group O, respectively. The estimate of the relative median potency for norepinephrine in group C versus group O was 0.643 (95% CIs, 0.363–0.956). The results of 95% CIs did not contain 1, demonstrating significant difference in norepinephrine requirement between the two groups. No significant difference among the groups in neonatal outcomes, such as umbilical artery blood gas analysis and Apgar scores (Table 3).

**Table 3:** Neonatal outcomes.

	Group O (*n*=75)	Group C (*n*=75)	*P*
Umbilical artery pH	7.36±0.02	7.36±0.02	0.811
Apgar score, 1 min	10 [9,10]	10 [9,10]	0.482
Apgar score, 5min	10 [10]	10 [10]	0.981
Birth weight (g)	3346±376	3332±353	0.817
Umbilical artery PCO_2_ (mmHg)	43.9±3.8	44.7±4.3	0.282
Umbilical artery PO_2_ (mmHg)	20.6±4.1	20.8±3.7	0.829
Umbilical artery HCO_3_ (mmol/l)	25.2±1.7	24.8±1.8	0.230

Data are presented as mean±SD or median [interquartile range].

## Discussion

In this dose-response study, the ED_50_ and ED_90_ of prophylactic infusion of norepinephrine combined with intravenous ondansetron were 0.021 (95% CIs, 0.013–0.029) µg/kg/min and 0.059 (95% CIs 0.044–0.089) µg/kg/min, respectively. The ED_50_ and ED_90_ of the saline control group were 0.033 (95% CIs, 0.024–0.043) µg/kg/min and 0.091 (95% CIs 0.068–0.147) µg/kg/min, respectively. The results revealed that the ED_50_ and ED_90_ of prophylactic norepinephrine infusion for the prevention of SAIH were reduced by approximately 36% and 35% with the use of intravenous 0.1 mg/kg ondansetron administered 10 min before spinal anaesthesia in parturients undergoing elective caesarean section.

Numerous studies have evaluated the effects of 5-HT3 receptor antagonists on SAIH, however, the results of these studies remain controversial^14,21,24,25^. Previous work from Aksoy *et al.*^24^ and El Khouly *et al.*^14^ concluded that administration of prophylactic ondansetron prior to spinal anaesthesia significantly reduced the incidence of SAIH and vasopressor consumption compared with placebo. In contrast, work from Marciniak *et al.*^25^ and Oofuvong *et al.*^21^ did not confirm these conclusions. The inconsistency of these research results may be related to the different dosages of ondansetron, timing of administration, definition of hypotension, dosage and type of spinal anaesthetic, and sample sizes. Consistent with two recently published meta-analyses^26,27^, our results indicated that prophylactic administration of ondansetron attenuated the incidence and severity of SAIH and vasopressor consumption in obstetric patients, although the difference was not statistically significant.

Unlike previous studies that focused on repetitive testing and sparse data, this is the first time that the interaction between norepinephrine and ondansetron has been studied in this manner. The strength of the current study was to compare the quantifiable effects of prophylactic intravenous ondansetron on norepinephrine requirement during spinal anaesthesia using a grouping method and to calculate the ED_50_ and ED_90_ values for the infusion dose of norepinephrine to provide a guide for the initial infusion dose of norepinephrine with or without prophylactic ondansetron intravenous injection.

A prospective study by Xiao *et al.*^16^ reported that intravenous injection of 4 mg ondansetron 10 min before spinal anaesthesia reduced the ED_50_ of prophylactic phenylephrine infusion in parturients undergoing caesarean section by approximately 26%, which was lower than the results of the current study but could be explained by the following points. First, the results may have been influenced by the interaction mechanism between different vasopressors and ondansetron. Second, variations in the study protocols could have led to different results. In our study, we calculated and compared the effective dose of norepinephrine infusion for preventing SAIH in each group using a grouping method, while they used up-down sequential analysis. The advantage of the grouping method with a relatively large sample size is that it ensures the balance of samples between each group, thereby controlling experimental errors and improving the reliability and accuracy of the experiment. Moreover, we not only calculated the ED_50_ of norepinephrine, but also determined the ED_90_ value. Because many anesthesiologists prefer to administer norepinephrine at a rate close to ED_90_ or ED_95_ in clinical practice. Third, compared with the fixed dose of ondansetron (4 mg) used in their study, the weight-based dose of ondansetron (0.1 mg/kg) used in the current study may maximize its effectiveness and prevent some parturients from experiencing overdose or suboptimal dose^24^, since obesity is becoming increasingly common among obstetric populations, and poses a growing challenge for obstetric anaesthesia^28,29^.

In addition to inhibition of the BJR, ondansetron has been reported to enhance the positive inotropic effect of norepinephrine due to the activation of pre-junctional and post-junctional 5-hydroxytryptophane-2A receptors or because of inhibition of norepinephrine reuptake to sympathetic nerve endings by activating imidazoline receptors^30^. The lower norepinephrine consumption in the ondansetron group observed in the present study may indicate this additional effect of ondansetron.

Accumulating evidence suggests that the correlation between organ damage and intraoperative hypotension is related to its duration and severity, with lower blood pressure leading to shorter exposure times for damage^31,32^ and also potentially resulting in neonatal acidosis^33^. Hence, it may be more clinically significant to intervene promptly in addressing the severity and duration of hypotension, rather than solely focusing on its incidence. In the current study, we conducted measurements of SBP at 1-minute intervals from spinal anaesthesia to delivery, with prompt intravenous intermittent rescue bolus of 6 µg norepinephrine once hypotension was detected. Thus, no adverse effects on neonatal outcomes were observed in both the groups.

Some studies have suggested that intravenous ondansetron might be associated with antagonizing the sensory block of intrathecal local anaesthetics, which may explain the weakened hemodynamic changes after spinal anaesthesia^34,35^. Nonetheless, we did not observe a significant difference in sensory block levels between the two groups. Thus, there is no evidence to indicate that the effect of the spinal anaesthesia level is a promoting mechanism. Previous studies have provided evidence that intravenous ondansetron significantly reduced the risks of bradycardia induced by spinal anaesthesia^10,26^. Nevertheless, bradycardia was observed in 1 parturient in group O versus 2 parturients in group C in the current study. This condition may be attributed to the mild β-adrenergic receptor agonist activity of norepinephrine, which counteracts the reflex slowing of HR. Moreover, the results of the Apgar score and cord blood analysis showed that prophylactic administration of ondansetron seemed to have no relevant impact on neonatal outcomes, which is consistent with the results of previous studies^36^.

We acknowledge several limitations in this study. First, although we have derived ED_50_ and ED_90_ values for norepinephrine in combination with or without prophylactic ondansetron. However, these values were estimated using probit regression rather than determining a 50% or 10% incidence of SAIH at these dose rates. Hence, it is necessary to verify and compare through clinical trials before they can be fully recommended in clinical practice. Second, the dosage of 0.1 mg/kg ondansetron used in this study might not be sufficient to maximize the prevention of BJR, it is reported that compared with the control group, a high dose of 0.15 mg/kg or 12 mg ondansetron could increase mean arterial pressure (MAP) in parturients during spinal anaesthesia for caesarean section^15,37^. The difference in norepinephrine requirements between the two groups might be more significant if prophylactic ondansetron was increased appropriately. However, we were concerned that the dosage of ondansetron above 0.15 mg/kg acting on 5-HT1B receptors might cause umbilical arterial vasoconstriction and be harmful to the foetus^38^. Third, in our study, ondansetron was administered 10 min before intrathecal injection, and the effect of different ondansetron administration times on the dose requirement of prophylactic norepinephrine needs to be further explored. Despite the fact that Qian *et al.*^39^ concluded that an earlier administration of prophylactic ondansetron 4 mg had no benefit in reducing the dose of prophylactic phenylephrine compared with a late administration, their study had a small sample size. Hence, a multicenter study with a larger sample size is required in the future. Fourth, we adopted SBP as a guiding parameter for blood pressure adjustments during caesarean section. Compared with SBP, MAP offers a more accurate reflection of placental perfusion and exhibits a stronger correlation with newborn prognosis^40^, thereby enhancing the scientific rigour and validity of the study.

## Conclusion

In conclusion, under the conditions of this study, intravenous ondansetron 0.1 mg/kg before spinal anaesthesia significantly reduced the dose requirement of prophylactic norepinephrine infusion in parturients undergoing elective caesarean section. This finding is potentially useful for clinical practice and further research.

## Ethical approval

This study was approved by the Institutional Review Board of Wenling Maternity and Child Health Care Hospital,Taizhou, China. (No. 2020-IRB-003) on November 9, 2020.

## Consent

Written informed consent was obtained from the patient for publication of this case report and accompanying images. A copy of the written consent is available for review by the Editor-in-Chief of this journal on request.

## Source of funding

This work was supported by funding from “Wenling Science and Technology Bureau (No. 2023S00164)” to “Zhejiang Medical and Health Science and Technology Plan Project (No. 2024XY101) and Wenling Science andTechnology Bureau (No. 2023S00164)”.

## Author contribution

Z.M.S., J.Q.M., and Z.M.: concept and design; Z.M.S.: drafting of the manuscript; Z.M.S., and H.Q.S.: statistical analysis; J.Q.M., J.L., and G.L.: acquisition, analysis, and interpretation of data; Z.M.S. and Z.M.: critical revision of the article; Z.M.S., H.Q.S., J.Q.M., J.L., G.L., and Z.M.: final approval of the article.

## Conflicts of interest disclosure

The authors declare that there are no conflicts of interest.

## Research registration unique identifying number (UIN)

1. Name of the registry: Chinese Clinical Trial Registry chictr.org.cn.
2. Unique Identifying number or registration ID: ChiCTR2000040295.
3. Hyperlink to your specific registration (must be publicly accessible and will be checked): https://www.chictr.org.cn/showproj.html?proj=64729.

## Guarantor

Zhi-min Sheng, Zhong Mei.

## Data availability statement

The data that support the findings of this study are available on request from the corresponding author on reasonable request.

## Provenance and peer review

Not commissioned, externally peer-reviewed.
